# Application of LC-MS/MS MRM to Determine Staphylococcal Enterotoxins (SEB and SEA) in Milk

**DOI:** 10.3390/toxins8040118

**Published:** 2016-04-20

**Authors:** Mirjana Andjelkovic, Varvara Tsilia, Andreja Rajkovic, Koen De Cremer, Joris Van Loco

**Affiliations:** 1 Food, Medicine and Consumer Safety, Scientific Institute of Public Health (WIV-ISP), Juliette Wytsmanstraat 14, 1050 Brussels, Belgium; varvara.tsilia@gmail.com (V.T.); koen.decremer@wiv-isp.be (K.D.C.); joris.vanloco@wiv-isp.be (J.V.L.); 2 Laboratory of Food Microbiology and Food Preservation, Ghent University (UGent), Coupure Links 657, 9000 Ghent, Belgium; andreja.rajkovic@ugent.be

**Keywords:** *Staphylococcus aureus* enterotoxins, SEA, SEB, milk, UPLC-ESI-MS/MS

## Abstract

*Staphylococcus aureus* is one of the important aetiological agents of food intoxications in Europe and can cause gastro-enteritis through the production of various staphylococcal enterotoxins (SEs) in foods. Due to their stability and ease of production and dissemination, some SEs have also been studied as potential agents for bioterrorism. Therefore, specific and accurate analytical tools are required to detect and quantify SEs. Online solid-phase extraction liquid chromatography electrospray ionization tandem mass spectrometry (online SPE-LC-ESI-MS/MS) based on multiple reaction monitoring (MRM) was used to detect and quantify two types of SE (A and B) spiked in milk and buffer solution. SE extraction and concentration was performed according to the European Screening Method developed by the European Reference Laboratory for Coagulase Positive Staphylococci. Trypsin digests were screened for the presence of SEs using selected proteotypic heavy-labeled peptides as internal standards. SEA and SEB were successfully detected in milk samples using LC-MS/MS in MRM mode. The selected SE peptides were proteotypic for each toxin, allowing the discrimination of SEA and SEB in a single run. The detection limit of SEA and SEB was approximately 8 and 4 ng/g, respectively.

## 1. Introduction

Enterotoxins produced by *Staphylococcus aureus* (SEs) are recognized as aetiologic agents of healthcare- and community-associated infection and food poisoning. The secreted toxins define the virulence of *S. aureus* although the arsenal of *S. aureus* virulence factors includes more than just toxins.

While *S. aureus* is heat sensitive, its enterotoxins are relatively heat stable (on an average 5 min at 56 °C [1]), making decontamination of a product heavily contaminated with SE difficult. Once formed in the food, the enterotoxins cannot be confidently eliminated. Furthermore, environmental conditions and food handling may also be a source of contamination. According to the available data, food intoxications due to *S. aureus* and its toxins are among the most common foodborne diseases [2].

SEs constitute a family of different serological types (SEA to SEE and SEG to SEU), which share similarities in structure, function and sequence [3,4]. The genes encoding the different enterotoxins are carried and disseminated by different mobile genetic elements, *i.e.*, prophages, plasmids, pathogenicity islands (SaPIs), enterotoxin gene clusters (*egc*) and the staphylococcal cassette chromosome (SCC). Enterotoxins are short, extracellular proteins that are water-soluble. They are most commonly described as very stable, and are resistant to heat as well as degrading enzymes. However, some cases have been reported where the toxins disappeared. Recently, SEA and SED have been found to decrease in boiled ham after a period of accumulation, and a number of earlier studies have reported the disappearance of SEA in broth, minced food and raw and pasteurized milk [5,6]. The reasons for this disappearance are not known yet, but one of the suggested hypotheses is an analytical artifact.

To date, more than 20 SEs or enterotoxin-like proteins (SE*l*s) have been identified and designated as SEA to typically SE*l*V [1,7]. Okumura *et al.* [8] reported also SE*l*W. Some of them are emetic agents (SEA to SEI, SER, SES, SET) and are a major cause of food poisoning [9]. While SEs are the toxins that induce emesis, the related SE*l*s either lack emetic activity or have not yet been tested for this [1]. Reported dose levels of staphylococcal enterotoxins involved with foodborne illness are scarce and varying, and the lowest suspected dose of SEA was about 0.5–0.75 ng/mL found in chocolate milk and infecting 850 school children [10]. Other reports suggested that SE-induced food poisoning can be caused by as little as 20–100 ng of enterotoxin consumed through food [11]. After ingestion, symptoms appear rapidly and abruptly, consistent with diseases caused by preformed toxins. The symptoms include copious vomiting, diarrhea, abdominal pain or nausea. Staphylococcal enterotoxins may be involved if *S. aureus* is present at levels of 5 log CFU/g and higher, however with lower counts reported to produce detectable SE amounts [12]. If the toxin is preformed and is present in the food prior to consumption, a sensitive detection of SE in food is imperative, given the consequences of staphylococcal foodborne poisoning and its current designation and potential use as a biological threat agent. Moreover, it is known that ingested *S. aureus* does not produce any (relevant) toxins in the human gastrointestinal tract [12].

Currently, the detection of *S. aureus* is based on determining a staphylococcus coagulase-positive reaction (and possibly catalase, hemolysis and thermonuclease). A mandatory SE detection is performed in case of suspected staphylococcal food poisoning. One of the validated methods is European Screening Method (ESM), already validated by the French Agency for Food, Environmental and Occupational Health & Safety (ANSES).

Existing detection methods for SEs include bio-assays, immunological assays, LC-MS or a combination of the mentioned techniques [13,14,15,16]. Alternatively, PCR detection of the enterotoxin coding genes can prove that a specific enterotoxin gene is present in the *S. aureus* isolated from the food source. However, this technique cannot prove a direct link between an SE and a food source, as food and processing factors affect the expression of enterotoxins. The most commonly used methods are different formats of immunological assays. Currently commercially available immunological methods for the detection of SEs as VIDAS-SET2 kit (enzyme-linked immunosorbent assay, ELISA methods), automated enzyme-linked fluorescent immunoassay (ELFA kit), reversed passive latex agglutination (SET-RPLA) do not detect all described enterotoxins, such as SEHwhich was involved in multiple food poisoning incidents [17] or toxic-shock syndrome toxin (TSST), or any other virulence-related proteins. Although immunological methods are sensitive and specific (e.g., the detection of SEB at <10 pg/mL by immuno quantitative PCR, Rajkovic *et al.*, [16]), the false positive and false negative reactions that are often found cannot be excluded through cross-reaction between serotypes or serological modification of the SEs due to food processing or toxin extraction, respectively. Furthermore, immunological assays can be costly and time-consuming due to the development of reliable and stable antibodies.

Recently, some novel methods using LC-MS/MS for the detection of whole toxins or their tryptic fragments have been developed [18,19,20,21,22,23]. However, various major drawbacks have prevented these methods from being implemented on a large scale. The main problem in LC-MS/MS-based methods is the extraction and purification of the protein toxins from a food matrix. The use of gels or lengthy immune-affinity purification is often required to get a sample clean and pure enough for LC-MS/MS analysis [18,20]. This technique allows us to identify and in some cases also quantify proteins, and assess changes in proteins due to different factors. The *S. aureus* proteome has recently become fully charted by means of a combination of high resolution and high mass accuracy of MS [24]. This in turn makes it possible to identify mechanisms involved in virulence, and map these mechanisms in different databases and even in stress/signatures libraries. Analytical improvement is necessary for protein separation, which is still mainly achieved by gel-based analysis. Further, the proteomic approach reveals the presence of actual toxins and not only the presence of their responsible activating genes as it is done in genomic studies. Therefore, there is a need for a sufficiently sensitive, efficient and quantitative proteomics-based method that will complement current SE detection methods.

In our laboratory, a method was developed with an online solid phase extraction, coupled to LC-MS/MS with detection in MRM mode (online SPE-LC-MS/MS-MRM), based on a bottom-up proteomic approach. It was tested in the scope of a large network project (*i.e.*, the EQuATox project—Establishment of Quality Assurance for the Detection of Biological Toxins of Potential Bioterrorism Risk). In this article, we will present the results obtained by analyzing the samples prepared for an EQuATox proficiency test. The goal of this work was to identify the presence or absence of SEB and SEA. Furthermore, for the samples which were found to contain SEB, we chose to apply stringent quality criteria for the acceptance of the results. The method has proven to be easily applicable to simple matrices (*i.e.*, low-protein or bacterial cultures), whereas the complexity of food rich in proteins, oxygen availability, salt content and pH still pose a challenge. The extraction from other matrices is still under optimization. This type of detection is a technically independent complementation of existing highly sensitive immunological methods, with the advantage of delivering unambiguous results. Its added value is that it is very specific and easy to develop and that it can be implemented in other food control laboratories with minimal requirements. The technique can also be adapted to detect other SE in foods or even other proteins of interests. Furthermore, with the use of certified standards as a reference, it has a great potential of being a quantitative method.

## 2. Results and Discussion

### 2.1. In Silico Selection of SEB Peptides

A UniProtKB search for “SEB AND Staphylococcus” generated more than 2000 hits. However, only one hit was directly assigned to SEB whereas the others were less specific or were denoted as fragments and were not further investigated. Out of 21 predicted tryptic peptides (obtained from an in silico digestion, Table S1) 6 were selected for synthetization (see experimental section). After optimization on the LC-MS/MS system, the three most intense peptides were used for the determination of SEB in the study (Table 1).

### 2.2. In Silico Selection of SEA Peptides

A UniProtKB search for “SEA AND Staphylococcus” generated 55 hits. Six of these hits were assigned directly to Staphylococcal enterotoxins type A, whereas the others were less specific or indicated as another enterotoxin type and were therefore not examined any further. Only two of the remaining sequences were reviewed (Swiss-Prot). Unreviewed sequences (TrEMBL) which were denoted as “Fragments” (Q6XZE9_STAAU, A0A097KJ70_STAAU, D0EMB3_STAAU, A0A097KJ81_STAAU) were removed from the analysis. The remaining two SEA sequences were aligned using Clustal Omega program. All sequences were identical (Figure S1).

The entry ETXA_STAAU was chosen to represent the most common SEA sequence encountered in the UniProtKB search. The 16 predicted tryptic peptides obtained from an in silico digestion are given in Table S2. From these peptides, five peptides were selected for synthetization (see experimental section). After optimization on the LC-MS/MS system, the two most intense peptides were further used in the study.

The following internal quality criteria for acceptance of the chromatographic results were used: (1) there could only be acceptance of the peaks with a signal-to-noise ratio above 3 (*S*/*N* > 3); (2) at least one of the selected proteotypic peptides was detected with both screened *m*/*z* transitions for the peptide; (3) the difference in retention time between the endogenous peptide and its internal standard was <0.03 min; and (4) the intensity rank of heavy peptides transitions had to match that of the endogenous peptides. Based on the *m*/*z* transitions for the labeled proteotypic peptides, we calculated the *m*/*z* transitions of the endogenous peptide fragments.

To verify the accuracy of the method, the study design included a blank reagent to determine the absence of any peaks and positive and negative control samples (uncontaminated and contaminated with a known SEB concentration). The final results showed that all reagent blanks were below the LOD. Neither SEB nor SEA were detected in any of negative control samples, and the results of the positive control material were satisfactory compared to those included in the study.

### 2.3. Toxin Detection

In total six blind samples were supplied in the proficiency testing (PT) and, as a result, six extracts (from buffer and milk, see Table 2) were prepared according to the setup explained below in the experimental section and internally assigned by codes. These codes were finally compared, and only after we had received the final report of the PT were the samples identified (Table 2). In the two buffer samples containing SEB at concentrations of 2 ng/g or below, no SEB was detected. This was in accordance with other results obtained by LC-MS/MS techniques in two other labs that participated in the PT. These labs used the same detection approach (mass detection) with a slightly different extraction procedure [26]. A possible reason for this is that the small spiked concentration is below the current detection limit of the LC-MS/MS methods. Nevertheless, the possibility of an analytical artifact might also be considered.

**Figure 1:**
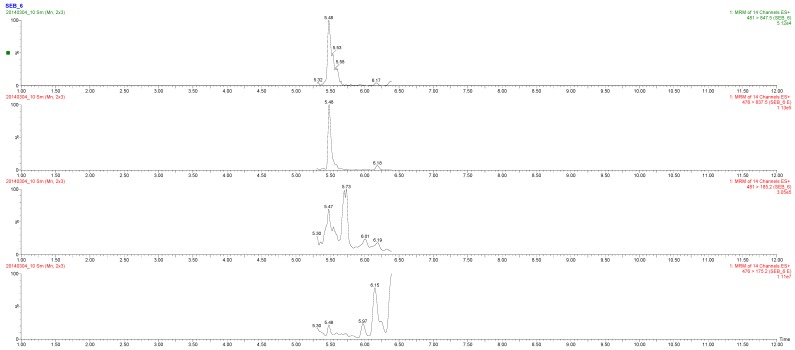
MRM-MS spectra from quantitative analysis of SEB in milk sample (M2). Abbreviation E (endogenous) stands for the peptides observed in the unknown samples.

**Figure 2:**
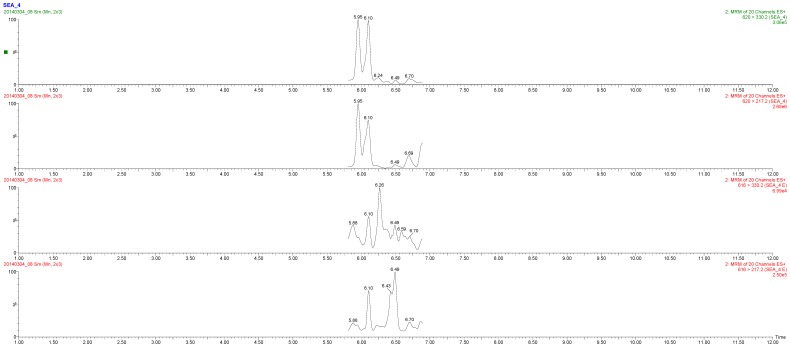
MRM-MS spectra from quantitative analysis of SEA in milk sample (M4). Abbreviation E (endogenous) stands for the peptides observed in the unknown samples.

In sample M1, only three very small peaks of SEB-2 at *m*/*z* 794.20 > 181.20; 794.20 > 213.30; 794.20 > 688.30 were seen with an *S*/*N* ratio < 3, resulting in a negative observation. In sample M2, the *m*/*z* peaks at 476.00 > 837.50 and 476.00 > 175.20 were correctly detected. The third designated *m*/*z* peak at 476.00 > 388.40 was not found. These peaks all correspond to the SEB6 peptide (Figure 1). Since only one of the three SEB peptides was detected, the M2 sample was tentatively reported to have a “possible SEB presence”. The concentration spiked in this sample was around 5 ng/mL, which is slightly above the LOD of the method. In sample M3, the presence of three peptides (SEB-2, SEB-5 and SEB-6) was observed. Whereas the latter two were observed with four expected peaks, the first resulted in only two. This was in accordance with the acceptance criteria and the sample was confidently determined as positive for SEB. Sample M4 was found not to contain SEB, but SEA (Figure 2). Both reference peptides were correctly detected according to all the criteria (table S3). The determined LODs for the method were established at 4 and 8 ng/g for SEB and SEA, respectively. These levels are comparable and even lower than most of those evaluated by Muratovic *et al.* [26]. This is comparable to the levels found in powdered skim milk (approx. 3.7 ng/g) in a Japanese outbreak [11]. Recently, a short literature overview has reported 30 references to *S. aureus* enterotoxins detected in milk [27]. These data were used to evaluate the consumption risk in China and suggested that the control of storage conditions during the period after heat treatment and before consumption would be key steps to minimize the risk of milk-borne staphylococcal intoxication. These pre-consumption points are also possible target points for applying the LC-MS/MS method toward ensuring food safety.

In this respect, protein standard absolute quantification (PSAQ) standards may also be used [20]. These are constituted protein standards which are very specific and may be used for absolute quantification. However, due to its high cost (to synthesize the constituted protein), this technique is not that attractive for contract laboratories which need fast, cheap and easily applicable methods to tackle routine food analysis. However, PSAQ might be a reliable method which still needs to be confirmed in further research.

The above results are based on the use of synthetic-labeled peptides of low purity grade. They are usually used for method evaluation and method setup. For a more precise and absolute quantification, AQUA grade standards are recommended [18]. Although these are more expensive standards (about 10 times higher in cost), they can be applied in routine analyses where the costs of one batch would be spread over thousands of samples, resulting in no more than one euro extra added cost per analyzed sample. The limitation of this preliminary study was the difficult calculation of the exact amount of the toxin due to the low purity grade of the synthetic peptides. This does not allow a complete evaluation of the method performance, including the recovery and digestion efficiency, but mainly underestimates the sensitivity of the method. Therefore, the use of the above-mentioned AQUA peptides will solve this problem in the future.

A recent report on the quantification of the SEA and SEB toxins uses similar technology [26]. The authors validated the method following an extensive validation design. In this study, however, only one peptide per toxin was used, NVTVQELDLQAR (SEA) and VTAQELDYLTR (SEB). The authors also briefly reviewed the current proteomic-based methodology used for the SE toxins. Among those mentioned and originally presented by our group at FoodMicro-2012 conference [20,21,22,23,28], the methods with low detection limit were developed in simple matrices or used whole proteins for detection. In contrast, a high protein matrix such as milk was scarcely investigated [29]. Our methods offer the advantage of unambiguous identification, provided that the detection limit can be driven to a point where natural contaminations are regularly found (pg/g). Nevertheless, immunological methods still have the advantage of being more sensitive. MS-based approaches may be able to improve this and detect SE down to a few ng/g after further improvement either in sample preparation strategies (size exclusion (SE), anion exchange (AE), strong cation exchange (SCX), or reversed-phase (RP) chromatography), or increasing proteolytic digestion efficiency or using nano-flow system. All of these would result in lower LODs and thereby being applicable to real samples.

The correct quantification analysis of mass spectrometry data is fundamental for the generation of reliable comparative data between different samples or replicates. Recently, a guideline was published for reporting quantitative mass spectrometry based experiments in proteomics [29]. This checklist outlines which information should be provided when reporting quantitative assays and they suggested to report all collected data. It currently does not include specific parameters like: how many peptides are needed to completely and confidently quantify and identify the protein, as is the case in regulated analyses control of contaminants in food [30]. Nevertheless, if the method has to be applied in food safety, food defense or other security issues, it is necessary to rigorously define the acceptance criteria per toxin [31]. Therefore, the guidelines that are routinely applied in food analyses (e.g., for veterinary residues, mycotoxin or pesticides) may be highly interesting, as well as more elaborate examples of how to proceed in analyzing suspected samples.

Besides LC-MS/MS techniques and immunoassays approaches for SE analyses, some other approaches were also described. Depending on whether the parameters were rapid and/or sensitive, some methods were applied at the level of 1 ng/mL, like for example biosensors linked to MS detection [32,33]. This method has a good sensitivity but still utilizes antibodies for which a specific production is needed. Nevertheless, it would be very useful to compare the two techniques as well as their applicability in everyday practices.

Finally, the method presented in this study, after further validation with AQUA peptides, may be applied for the direct identification of SEA and SEB. The identification may complement the current screening/semi-quantitative post intoxication approach in samples that were suspected to induce illness in humans or by identifying toxin on the serotype level in pre-market food samples as part of the food control programs.

## 3. Experimental Section

### 3.1. Samples

As part of the EQuATox project, we obtained six blind extracts (four milk samples and two buffer extracts). One of the four milk samples, was not contaminated with SEB or SEA and was used as a blank sample (M1). The other three milk samples were spiked at three different levels with either SEB or SEA: milk spiked with SEB at 5 ng/g (M2), with SEB at 25 ng/g (M3), and with SEA at 10 ng/g (M4). These samples were prepared in the context of the proficiency testing (PT) organized by FrenchFood Safety Agency, in particular the European Reference Laboratory for Coagulase Positive Staphylococci (ANSES, Paris, France). Two samples marked B1 and B2 were two PBS/BSA buffered samples containing a lower amount of SEB (0.5 ng/mL and 2 ng/mL, respectively).

The samples were prepared to evaluate the specificity of the methods used by the participating laboratories. The SEB contamination levels were selected after evaluating the quantification limits (LOQ) as reported by the participant prior to the PT. Some of those levels were lower than those currently reported for LC-MS/MS detection.

### 3.2. Sample Preparation

**Figure 3:**
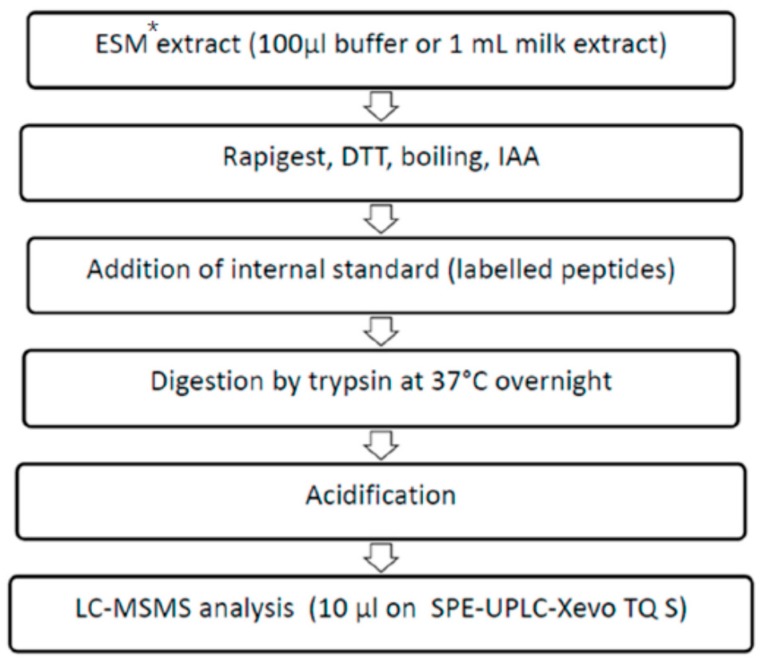
Study protocol scheme whereby the samples were analysed by LC-MS/MS. The samples originated from the proficiency test (PT) organized in the scope of EQuATox project. * ESM: European Screening Method recommended by EURL for Coagulase Positive Staphylococci. The details of the method are explained in [15].

Milk samples (M1, M2, M3 and M4) were previously subjected to the extraction according to the the so-called European Screening Method (ESM) based on extraction, dialysis concentration and qualitative immunochemical detection has to be applied for analysis [15]. These extracts were subsequently used for analysis by the VIDAS and PCR test, whereas only 100 µL (buffer solutions B1 and B2) and 1 mL (milk samples, M1-4) were used further. Those obtained extracts (*i.e.*, partially purified milk) were dried under nitrogen flow and resuspended in 100 µL of NH_4_HCO_3_. 10 µL 1% Rapigest SF (Waters, Zellik, Belgium) and 10 µL 100 mM DTT (prepared in 200 mM 50 mM NH_4_HCO_3_) were added to these 100 µL of sample or in 100 µL buffer and were boiled for 10 min. After they had cooled down to room temperature and reacted with IAA, the samples were spiked with proteotypic peptides of SEA and SEB (Thermo Fisher Scientific, Ulm, Germany) and digested with modified trypsin (Promega). Digestion took place overnight at 37 °C. Trypsin activity was quenched by adding 10 µL of 100% HCOOH (Merck Chemicals N.V./S.A, Overrijse, Belgium). The analytical protocol is depicted in Figure 3.

### 3.3. Stock Solutions and Reagents

All chemicals and reagents were obtained from commercial vendors (*i.e.*, Staphylococcal enterotoxins A and B, Iodoacetamide (IAA, BioUltra), Dithiothreitol (DTT, BioXtra), and Urea (BioReagent) from Sigma-Aldrich (Bornem, Belgium)). Methanol (HPLC grade) and formic acid (UPLC-MS grade) were obtained from Biosolve BV (Valkenswaard, The Netherlands). Ammonium acetate (TraceSELECT^®^) came from Sigma-Aldrich (Bornem, Belgium). The HPLC grade water was produced by means of a Milli-Q^®^ Gradient A10 water purification system (Millipore, Bedford, MA, USA).

The synthetic-labeled internal standard peptides VLYDDNHVSAINVK, VTAQELDYLTR, LGNYDNVR, YNLYNSDVFDGK and SELQGTALGNLK were custom-made by Thermo-Scientific (Ulm, Germany) as crude preparations of FasTrack 1. The heavy label was on the *C*-terminal K* (U-13C6/15N2) or R* (U-13C6/15N4) and resulted in a 8 and 10 kDa mass difference, respectively, compared to the corresponding endogenous peptides.

### 3.4. In Silico Selection of Peptides

UniProt release 2014_09 was used to obtain SEA and SEB sequences. The database UniProt Knowledgebase (UniProtKB, Magrane and the UniProt consortium, 2011) was searched for the terms “SEA (or SEB) AND Staphylococcus”. Multiple sequence alignment was performed using Clustal O v. 1.2.1 [34].

Consequently, the selected protein was in silico digested using PeptideMass (Expasy). The chosen enzyme was trypsin, the cysteines were treated with iodoacetamide and no missed cleavages were allowed. Peptide masses were calculated based on the monoisotopic masses of the amino acid residues.

In order to determine the specificity of the candidate proteotypic peptides, sequences with more than six amino acid residues were blasted with BLASTP 2.2.30+ in NCBI using the default parameters. Proteotypic peptides are peptides that can be used to uniquely identify a specific protein [35]. If peptides were also found to be present in other SEs or in other proteins, such as those from human origins, these were not selected.

Proteotypic SEB and SEA peptides with amino acids susceptible to side reactions, such as oxidation of W, M and C [36], were not selected. Peptides with internal P or G were favored as they are known to generate intense fragments. The average hydrophilicity of the remaining peptides was calculated using Bachem’s Peptide Calculator [37] Very hydrophobic (highly negative) and very hydrophilic (highly positive) peptides were rejected.

### 3.5. Internal Standards (IS)

An initial panel of 11 specific tryptic peptides was selected for synthetization and further optimization with MRM. These peptides followed the selection rules for MRM assay development and it was verified that they were free from synthesis and transformation issues.

A digest IS to determine the digestion efficiency and act as positive control for digestion was designed based on two AA substitutions in the both SEB_2 and SEA_2. The two resulting peptide sequences were added together to form VTAQE**VE**YLT**R**YNLYN**TE**VFDGK.

Internal standard stock solutions of 50 pmol/µL were made by dissolving the IS in a 1:1 mixture of MilliQ water and acetonitrile. These stock solutions were then further used to make the IS mix containing all IS for each toxin at a final concentration of 10 pmole/µL.

### 3.6. LC-MS/MS Analysis

After digestion and quenching, the samples were centrifuged at 13,000 rpm for 2 min and the supernatant was transferred to UPLC vials. The LC-MS/MS system consisted of an UPLC-MS/MS Waters Xevo TQ-S system (Waters, Zellik, Belgium) with electrospray ionization (ESI) source coupled with an online solid phase extraction device. A 10 µL (full loop) sample was injected. An Oasis HLB 2.1 mm × 20 mm column (Waters) was used in combination with an Acquity BEH C18 2.1 mm × 100 mm, 1.7 µm (Waters) column at 40 °C as trapping and analytical columns, respectively.

The mobile phases consisted of solution A (0.1% HCOOH in acetonitrile) and solution B (0.1% HCOOH in milli-Q). During the first 2 min, isocratic conditions at 100% B were used at a 1 mL/min flow. After the SPE-valve was switched, a linear gradient elution was performed from 0% to 100% A in 4.5 min. Finally, the column was washed and re-equilibrated for 3 min at 100% B. Injection to injection time was 15 min.

## 4. Conclusions

The analytical technique LC combined with tandem MS using multiple reaction monitoring (MRM) is regarded to be highly specific. In the present study, a proteomics-based bottom-up approach was used to identify SEA and SEB in the milk and buffer solution. Our results show that this kind of method has the potential to be practically used in food control or security applications, as long as the strict quality criteria that are normally applied for this kind of analytical method are met. The obtained detection limits are acceptable, thus making further optimization for different food matrices possible.

## Figures and Tables

**Table 1:** Summary of tryptic peptides of SEB and SEA selected and evaluated for their use in the UPLC-ESI-MS/MS analysis.

Toxin	Peptide Sequence	Peptide Study Code *	Position	*C*-End	Peptide Mass	*Q*1 > *Q*2 *m*/*z* **	Cone Voltage (V)	Collision Energy (eV)
SEA	YNLYNSDVFDGK	SEA-2	191–202	K	1434.654	722.0 > 474.40	25	33
						722.0 > 391.30	25	23
						722.0 > 278.20	25	23
						722.0 > 212.20	25	23
	SELQGTALGNLK	SEA-4	40–51	K	1230.669	620.0 > 330.20	25	22
						620.0 > 217.20	25	20
SEB	VLYDDNHVSAINVK	SEB-2	53–66	K	1586.817	798.2 > 692.30	25	15
						798.2 > 213.30	25	15
						798.2 > 185.20	25	31
						532.6 > 185.20	25	31
	VTAQELDYLTR	SEB-5	182-192	R	1308.679	660.0 > 562.50	25	30
						660.0 > 919.60	25	20
						660.0 > 790.60	25	20
						660.0 > 677.50	25	20
	LGNYDNVR	SEB-6	85-92	R	950.469	481.0 > 847.50	25	15
						481.0 > 398.40	25	21
						481.0 > 185.20	25	15

* Selected predicted peptide sequences of SEB (ETXB_STAAU) and SEA (ETXA_STAAU) after in silico digestions using PeptideMass software [25]. Only tryptic peptides (*C*-terminal K or R) without any missed cleavages are given. Mass is the monoisotopic mass of the uncharged peptide. ** *Q*1, quantifier and *Q*2 qualifier ion.

**Table 2:** Qualitative results of the tested samples obtained by LC-MS/MS and following the modified criteria as explained in EU 2002/657.

Sample (Matrix)	Test Portion Used (mL or g)	SEA Observed	SEB Observed	Peptide Observed (Study Code)	Actual Toxin Presencse *
B1 buffer sample spiked at	100 µL	-	-	no	0.5 ng SEB/g
B2 buffer sample spiked at	100 µL	-	-	no	2 ng SEB/g
M1 (milk)	1 mL extract	-	-	no	No
M2 (milk)	1 mL extract	-	±	SEB-6	5 ng SEB/g
M3 (milk)	1 mL extract	-	+	SEB-2	25 ng SEB/g
				SEB-5	
				SEB-6	
M4 (milk)	1 mL extract	+	-	SEA-4	10 ng SEA/g
				SEA-2	

* The detection limit (LOD) of SEA and SEB was approximately 8 and 4 ng/g, respectively.

## Supplementary Files

Supplementary File 1
