# Supplementary material for: Application of LC-MS/MS MRM to Determine Staphylococcal Enterotoxins (SEB and SEA) in Milk
Source: Toxins (Basel). 2016 Apr 20;8(4):118. doi: 10.3390/toxins8040118 (PMC4848643; doi:10.3390/toxins8040118)
Supplement: Supplementary File 1 [file toxins-08-00118-s001.pdf]

Supplementary Materials: Applcation of LC-MS/MS MRM to Determine Staphylococcal Enterotoxins (SEB and SEA) in Milk

Mirjana Andjelkovic, Varvara Tsilia, Andreja Rajkovic, Koen De Cremer and Joris Van Loco

Table S1. Predicted peptide sequences of SEB (ETXB_STAAU) after *in silico* digestions using PeptideMass software. Only tryptic peptides (*C*-terminal K or R) without any missed cleavages are given. Mass is the monoisotopic mass (not including AM) of the uncharged peptide [M]. AM: Artificial modification.

Order number	Mass	Position	AM	Peptide Sequence	Length (number of amino acids)
1	2290.978	216–234	-	FIENENSFWYDMMPAPGDK	19
2	2129.931	108–124	Cys_CAM: 120	YVDVFGANYYYQCYFSK	17
3	1866.811	139–155	Cys_CAM: 140	TCMYGGVTEHNGNQLDK	17
4	1864.973	67–81	-	SIDQFLYFDLIYSIK	15
5	1837.864	201–215	-	LYEFNNSPYETGYIK	15
6	1586.817	53–66	-	VLYDDNHVSAINVK	14
7	1519.739	28–40	-	ESQPDPKPDELHK	13
8	1308.679	182–192	-	VTAQELDYLTR	11
9	1278.669	169–179	-	NLLSFDVQTNK	11
10	1272.582	126–136	-	TNDINSHQTDK	11
11	1191.517	240–248	-	YLMMYNDNK	9
12	1070.501	44–52	-	FTGLMENMK	9
13	966.5506	257–264	-	IEVYLTTK	8
14	950.469	85–92	-	LGNYDNVR	8
15	694.3406	163–168	-	VFEDGK	6
16	659.3875	193–197	-	HYLVK	5
17	624.2987	235–239	-	FDQSK	5
18	579.2807	249–253	-	MVDSK	5
19	575.3511	158–162	-	SITVR	5
20	561.2878	99–103	-	DLADK	5
21	522.2922	93–96	-	VEFK	4

Table S2. Predicted peptide sequences of SEA (ETXA_STAAU) after in silico digestions using PeptideMass software. Only tryptic peptides (*C*-terminal K or R) without any missed cleavages are given. Mass is the monoisotopic mass (not including AM) of the uncharged peptide [M]. AM: Artificial modification.

Order number	Mass	Position	AM	Peptide Sequence	Length (number of amino acids)
1	3327.664	206–235	-	GLIVFHTSTEPSVNYDLFGA QGQYSNTLLR	30
2	2306.04	80–98	-	GFFTDHSWYNDLLVDFDSK	19
3	2039.917	109–127	Cys_CAM: 120	VDLYGAYYGYQCAGGTPNK	19
4	1913.895	242–257	-	TINSENMHIDIYLYTS	16
5	1742.886	66–79	-	ESHDQFLQHTILFK	14
6	1651.732	128–142	Cys_CAM: 130	TACMYGGVTLHDNNR	15
7	1434.654	191–202	-	YNLYNSDVFDGK	12
8	1385.738	173–184	-	NVTVQELDLQAR	12
9	1230.669	40–51	-	SELQGTALGNLK	12
10	1154.657	149–158	-	VPINLWLDGK	10

Order number	Mass	Position	AM	Peptide Sequence	Length (number of amino acids)
11	1128.626	159–168	-	QNTVPLETVK	10
12	1120.531	52–59	-	QIYYYNEK	8
13	848.3996	28–34	-	SEEINEK	7
14	680.3613	186–190	-	YLQEK	5
15	619.3297	143–147	-	LTEEK	5
16	589.3191	99–103	-	DIVDK	5

Table S3. Quality criteria used for acceptance of the LC-MS/MS results.

Criteria	Accepted Limits or Descriptions
Retention time	RRT = RT of the IS ± 0.05
Mass spectral data criteria (Mention if any standards are followed)	According to EU-2002-657
Other applied criteria	1. The *S*/*N* * should be higher than 3<br>2. Visual control of chromatograph<br>3. At least one positive proteotypic peptide<br>4. Retention time of light peptides = Retention of heavy peptides ±0.05 min<br>5. 2 transitions positive per proteotypic peptide<br>6. The intensity rank of the transitions of heavy peptides should match the intensity rank of the transitions of endogenous peptides

* *S*/*N*, signal to noise ratio.

```
SP|P0A0L2|ETXA_STAAU
MKKTAFTLLLFIALTLTTSPLVNGSEKSEEINEKDLRKKSELQGTALGNLKQIYYYNEKA 60
SP|P0A0L1|ETXA_STAAW
MKKTAFTLLLFIALTLTTSPLVNGSEKSEEINEKDLRKKSELQGTALGNLKQIYYYNEKA 60
************************************************************

SP|P0A0L2|ETXA_STAAU
KTENKESHDQFLQHTILFKGFFTDHSWYNDLLVDFDSKDIVDKYKGKKVDLYGAYYGYQC 120
SP|P0A0L1|ETXA_STAAW
KTENKESHDQFLQHTILFKGFFTDHSWYNDLLVDFDSKDIVDKYKGKKVDLYGAYYGYQC 120
************************************************************

SP|P0A0L2|ETXA_STAAU
AGGTPNKTACMYGGVTLHDNNRLTEEKKVPINLWLDGKQNTVPLETVKTNKKNVTVQELD 180
SP|P0A0L1|ETXA_STAAW
AGGTPNKTACMYGGVTLHDNNRLTEEKKVPINLWLDGKQNTVPLETVKTNKKNVTVQELD 180
************************************************************

SP|P0A0L2|ETXA_STAAU
LQARRYLQEKYNLYNSDVFDGKVQRGLIVFHTSTEPSVNYDLFGAQGQYSNTLLRIYRDN 240
SP|P0A0L1|ETXA_STAAW
LQARRYLQEKYNLYNSDVFDGKVQRGLIVFHTSTEPSVNYDLFGAQGQYSNTLLRIYRDN 240
************************************************************

SP|P0A0L2|ETXA_STAAU KTINSENMHIDIYLYTS 257
SP|P0A0L1|ETXA_STAAW KTINSENMHIDIYLYTS 257
```

Figure S1. Alignment of the SEA sequences using CLUSTAL O (1.2.1). This program aligned two selected ETXA_STAAU and ETXA_STAAW protein sequences delivering accurate alignments.
